# Protein expression of the tear film of domestic cats before and after inoculation with *Toxoplasma gondii*

**DOI:** 10.1186/s12917-021-03080-9

**Published:** 2021-12-14

**Authors:** Paula Elisa Brandão Guedes, Jéssica Fontes Veloso, Luciana Carvalho Lacerda, Juliano Oliveira Santana, Irma Yuliana Mora-Ocampo, Carlos Priminho Pirovani, Rebeca Dalety Santos Cruz, Alexandre Dias Munhoz, Renata Santiago Alberto Carlos

**Affiliations:** 1 Santa Cruz State University, Rodovia Jorge Amado, km 16, Salobrinho, Bahia Ilhéus, Brazil; 2 Federal University of Western Bahia, Av. 23 de Agosto, S/N, Assunção, Bahia Barra, Brazil; 3 Department of Biological Sciences, Santa Cruz State University, Ilhéus, Brazil; 4 Department of Agricultural and Environmental Sciences, Santa Cruz State University, Ilhéus, Brazil

**Keywords:** *Felis catus*, Proteome, Teardrop, Toxoplasmosis

## Abstract

**Background:**

Tear film (TF) helps maintain and protect ocular function against damage to the ocular surface. Proteins are one of its main constituents, whose expression pattern can be used as a biomarker of ocular changes and systemic diseases. The aim of this study was to evaluate the expression of proteins in the TF of domestic cats before and after infection with *Toxoplasma gondii*, in the phases of acute infection and chronicity. Twelve healthy cats received orally homogenized brain matter obtained from mice inoculated with *T. gondii* oocysts, strain ME49. Cat feces were collected daily from the third day after infection to assess the release of oocysts. TF samples were obtained from cats, by Schirmer’s Tear Test 1, on day 0 (before infection), day 5 after infection (acute phase of infection, with maximum peak release of oocysts in feces) and on day 21 after infection (start of chronic phase, 7 days after total absence of oocyst release in feces). Tear samples were also submitted to proteomic analysis in a Q-Tof-Premier mass spectrometer.

**Results:**

A total of 37 proteins with scores equal to or greater than 100 were identified on D0, followed by 36 on D5 and 42 on D21. Of these, 27 were common to D0 and D5, 33 to D0 and D21, 27 to D5 and D21, and 26 were common to the three groups, totaling 54 proteins. The most abundant proteins were lipocalin allergen Fel d, serum albumin, aldehyde dehydrogenase, lactoperoxidase and lactotransferrin. There was no significant difference in the abundance of proteins found on D0 and D5, but there was a statistical difference between D0 and D21 for ACT1_AEDAE, CERU_HUMAN and GELS_HUMAN. Regarding D5 and D21, there were significant differences for KV1_CANLF, LAC_PIG, TRFL_PIG, ACT1_AEDAE, CERU_HUMAN, GELS_HUMAN and OVOS2_HUMAN.

**Conclusions:**

The main proteins identified in the TF of domestic cats are similar to those found in humans and other animal species. Most are part of the ocular surface defense system against injuries. The most expressed proteins in animals in the chronic phase of *T. gondii* infection are associated with the immune response to the parasite.

**Supplementary Information:**

The online version contains supplementary material available at 10.1186/s12917-021-03080-9.

## Background

Tear film (TF) is a viscous, trilaminar fluid that forms an interface between ocular tissue and air and consists of layers of components that cover the surface of the eyes, composed of proteins, lipids, water and electrolytes, which protect the ocular surface [1, 2]. The ocular surface, in turn, is constantly exposed to external and internal factors that can generate changes in its homeostasis, such as ultraviolet radiation and environmental pollutants, which generate oxidative stress [3], in addition to ocular and systemic pathologies [2]. In this context, the TF is of fundamental importance for the maintenance of a healthy ocular surface, as well as for defense against damage. Thus, the interaction between the TF and the corneal and conjunctival epithelium are crucial to maintain the ocular protective barrier [3, 4]. Among its main functions are lubrication of the eyelids, conjunctiva and cornea; nutrition of the cornea through the transport of nutrients and metabolites to its surface; removal of foreign bodies from the conjunctiva and cornea; and maintenance of the surface for light refraction and defense against pathogens and harmful substances [1]. Therefore, tissue constituents such as proteins are essential for the functioning of these mechanisms [2].

Studies involving TF components, especially proteins, can help in the assessment of tear production abnormalities associated to ocular changes, such as in cases of keratoconjunctivitis [5]. Furthermore, research indicates that changes in the expression of proteins in the TF can also accompany systemic diseases, since proteins play an important role in combating pathogens, as mentioned above [2].

Toxoplasmosis is a widely studied systemic disease with significant importance for human and animal health. This is a zoonosis with worldwide distribution, caused by the protozoan *Toxoplasma gondii*, of which felids are the definitive hosts. Depending on the stage of infection, the disease can have different clinical signs in cats, such as depression, anorexia, jaundice, dyspnea, convulsions and ocular changes such as uveitis [6].

Although molecular studies of aqueous humor and tear samples have already been developed in human [7–9] and veterinary medicine [2, 10–16], only one study on the tear proteome of domestic felines has been published so far [17], carried out with healthy cats. Thus, the objective of this study was to evaluate the expression of proteins in the TF of healthy domestic cats before and after inoculation with *Toxoplasma gondii*, in the acute phase and start of the chronic phase of the infection.

## Results

**Fig. 1:**
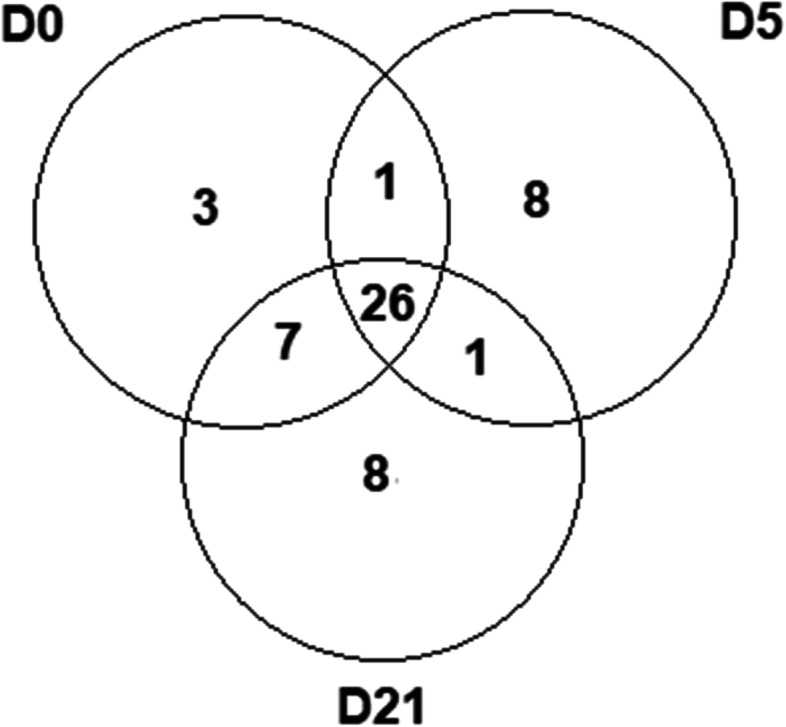
Venn diagram illustrating the number of proteins found in the TF of domestic cats, through mass spectrometry, in the different sample groups. Source: personal file

A total of 37 proteins with scores equal to or greater than 100 were identified on D0, while 36 were noted on D5 and 42 on D21. Of these, 27 were common to D0 and D5, 33 to D0 and D21, 27 to D5 and D21, and 26 were common to the three groups, thus totaling 54 proteins (Fig. 1).

**Fig. 2:**
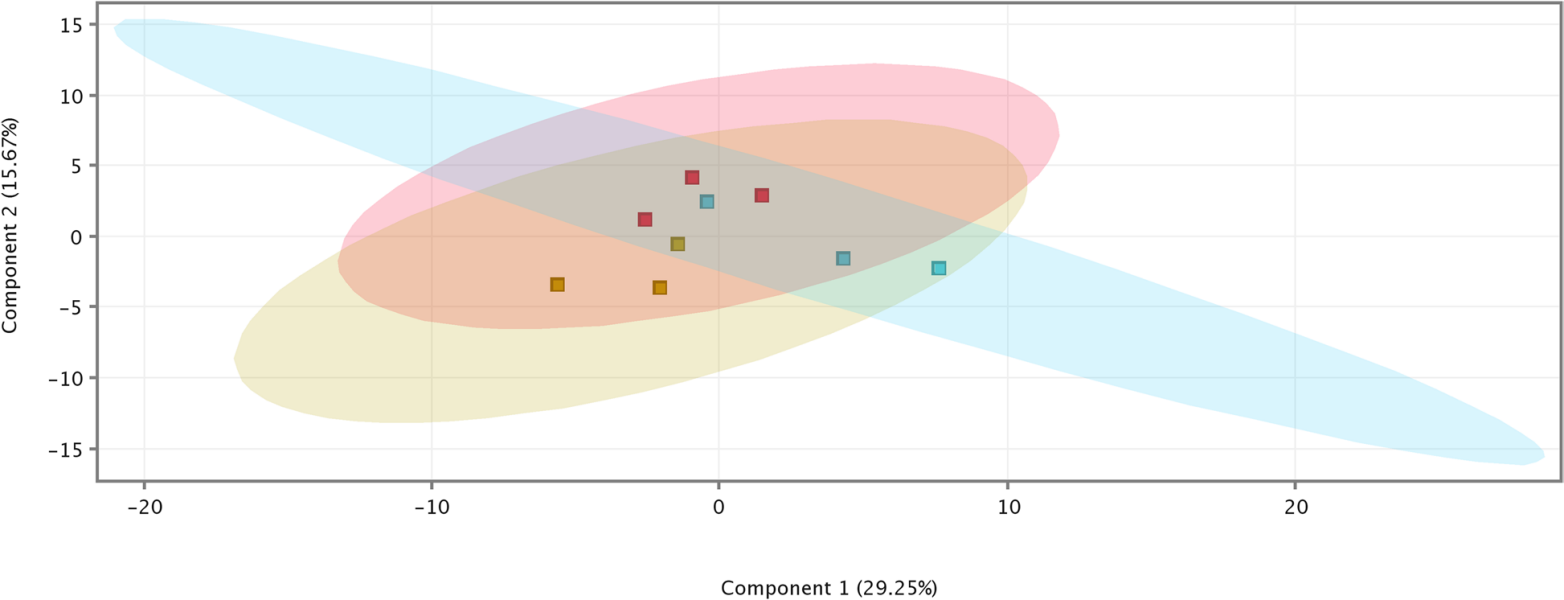
Principal Component Analysis (PCA) Graph. Subtitle:  D0;  D5;  D21. Source: personal file

The PCA graph (Fig. 2) revealed that the accumulation of proteins expressed on D21 was grouped according to the day of collection, also indicating a distinct diversity in relation to day D0 and a proximity to day D5.

The proteins that predominated in the TF of healthy domestic cats were allergen feld 4 (lipocalin), serum albumin, aldehyde dehydrogenase, lactoperoxidase and lactotransferrin and others, which are identified in Table 1. In addition, the following were observed in cats after infection with *T. gondii*: malate dehydrogenase, serotransferrin, keratin, phosphoglycerate mutase, elongation factor, ceruloplasmin, gelsolin, crystalline alpha chain and annexin A1.

**Table 1:** Proteins identified in the tear of healthy and infected cats

Compound name	SWISS-PROT ID	Mass (Da)	Score/Peptide matches								
			D0a	D0b	D0c	D5a	D5b	D5c	D21a	D21b	D21c
Allergen Fel d 4 OS=*Felis catus* PE = 1 SV = 1	ALL4_FELCA	21,580	11,292/346	14,539/383	12,265/350	12,427/316	6378/186	1968/92	9444/ 245	11,693/283	11,127/302
Serum albumin OS=*Felis catus* GN = ALB PE = 1 SV = 1	ALBU_FELCA	70,611	2740/76	4467/119	3544/93	2867/71	2245/61	933/30	3460/89	3646/93	2601/65
Aldehyde dehydrogenase, dimeric NADP-preferring OS=*Canis lupus familiaris* GN = ALDH3A1 PE = 2	AL3A1_CANLF	51,193	509/17	731/23	598/17	779/26	647/16	266/7	842/23	775/24	606/20
Lactoperoxidase OS=*Bos taurus* GN = LPO PE = 1	PERL_BOVIN	81,504	427/18	783/28	562/19	1031/36	535/17	299/9	586/23	684/23	585/21
Lactotransferrin OS=*Bos taurus* GN = LTF PE = 1 SV = 2	TRFL_BOVIN	80,002	410/15	667/27	534/19	643/20	604/23	283/18	542/18	699/21	450/17
Lactotransferrin (Fragment) OS = *Equus caballus* GN = LTF PE = 1 SV = 1	TRFL_HORSE	77,938	184/7	238/11	283/12	275/8	425/14	189/6	237/9	343/11	184/8
Inhibitor of carbonic anhydrase OS=*Sus scrofa* GN=ICA PE = 1	ICA_PIG	79,636	406/10	471/10	375/6	877/17	911/20	187/4	695/15	777/16	514/10
Major allergen I polypeptide chain 1 OS=*Felis catus* GN=CH1 PE = 1 SV = 2	FEL1A_FELCA	10,364	353/7	781/12	709/9	725/13	ND	ND	219/3	746/11	487/9
Trypsin OS=*Sus scrofa* PE = 1 SV = 1	TRYP_PIG	25,078	255/7	260/11	289/8	459/13	196/8	184/6	255/8	527/12	212/6
Actin, plasmodial isoform OS=*Physarum polycephalum* GN = ARDA PE = 1	ACTA_PHYPO	42,001	227/10	ND	ND	ND	ND	ND	ND	ND	ND
Deleted in malignant brain tumors 1 protein OS=*Oryctolagus cuniculus* GN=Dmbt1 PE = 1	DMBT1_RABIT	177,328	201/4	293/5	259/4	504/9	327/6	276/7	393/7	167/3	ND
Haptoglobin OS = *Ateles geoffroyi* GN=HP PE = 2	HPT_ATEGE	38,964	200/11	ND	ND	ND	ND	ND	ND	ND	ND
Haptoglobin OS=*Canis lupus familiaris* GN=HP PE = 1	HPT_CANLF	36,890	128/7	198/7	122/3	265/10	129/5	103/5	218/9	217/23	106/7
Glutathione S-transferase P OS=*Bos taurus* GN = GSTP1 PE = 1	GSTP1_BOVIN	23,826	190/4	303/6	375/7	407/5	116/2	ND	289/4	573/8	282/4
Ig kappa chain V region GOM OS=*Canis lupus familiaris* PE = 1 SV = 1	KV1_CANLF	12,112	158/2	428/6	178/2	ND	ND	ND	205/3	429/5	324/4
Ig lambda chain C region OS=*Sus scrofa* PE = 1	LAC_PIG	11,168	155/8	435/18	361/15	105/4	ND	ND	241/12	421/16	347/11
Polymeric immunoglobulin receptor OS=*Bos taurus* GN=PIGR PE = 2	PIGR_BOVIN	83,695	150/6	126/6	118/5	ND	106/6	ND	224/12	239/8	ND
Ovostatin homolog 2 OS=*Homo sapiens* GN=OVOS2 PE = 2 SV = 2	OVOS2_HUMAN	162,517	135/5	271/9	208/6	329/12	316/14	302/15	216/8	226/9	212/8
Retinal dehydrogenase 1 OS=*Oryctolagus cuniculus* GN = ALDH1A1 PE = 1	AL1A1_RABIT	54,933	110/2	ND	ND	ND	ND	ND	ND	ND	142/4
Ovostatin homolog OS = *Mus musculus* GN=Ovos PE = 2 SV = 2	OVOS_MOUSE	163,607	108/6	163/11	ND	161/9	215/11	208/11	150/9	162/10	124/7
Immunoglobulin heavy variable 3–15 OS=*Homo sapiens* GN=IGHV3–15 PE = 3	HV315_HUMAN	13,089	105/4	239/12	112/7	115/4	178/6	102/4	214/8	ND	108/5
Glutathione S-transferase P OS=*Bos taurus* GN = GSTP1 PE = 1	GSTP1_CRILO	23,851	102/2	198/5	155/4	ND	160/4	ND	174/4	170/4	ND
Lactotransferrin OS=*Camelus dromedarius* GN = LTF PE = 1	TRFL_CAMDR	79,158	ND	608/22	509/17	725/19	ND	ND	ND	746/23	ND
Actin-10 OS=Dyctiostelium discoideum GN = act10 PE = 1 SV = 1	ACT10_DICDI	41,948	ND	312/15	ND	ND	ND	ND	ND	342/23	368/19
Actin, muscle-type OS = *Molgula oculata* PE = 3 SV = 1	ACT2_MOLOC	42,521	ND	274/13	ND	ND	ND	ND	487/17	ND	ND
Aldehyde dehydrogenase, cytosolic 2 (Fragment) OS = *Macroscelides proboscideus* PE = 2	ALDH2_MACPR	26,841	ND	274/10	370/9	ND	ND	ND	ND	207/5	ND
Haptoglobin OS=*Bos taurus* GN=HP PE = 2 SV = 1	HPT_BOVIN	45,629	ND	231/12	158/7	ND	288/17	253/14	186/12	180/10	118/10
Selenium-binding protein 1 OS = *Rattus norvegicus* GN=Selenbp1 PE = 1 SV = 1	SBP1_RAT	53,069	ND	256/9	ND	177/8	ND	ND	297/11	ND	ND
Alpha-enolase OS=*Homo sapiens* GN = ENO1 PE = 1 SV = 2	ENOA_HUMAN	47,481	ND	220/8	179/5	173/7	ND	ND	163/6	151/6	177/6
14–3-3 protein sigma OS=*Bos taurus* GN=SFN PE = 2	1433S_BOVIN	27,946	ND	133/10	119/3	172/6	140/7	ND	325/14	236/12	194/7
Malate dehydrogenase OS = *Myxococcus xanthus* GN = mdh PE = 3 SV = 1	MDH_MYXXA	33,194	ND	120/14	ND	101/16	ND	ND	ND	106/19	118/14
Serotransferrin OS = *Mus musculus* GN = Tf PE = 1 SV = 1	TRFE_MOUSE	78,841	ND	109/6	ND	108/6	141/7	134/7	133/6	105/6	ND
14–3-3 protein zeta/delta OS=*Bos taurus* GN=YWHAZ PE = 1 SV = 1	1433Z_BOVIN	27,899	ND	ND	104/3	ND	ND	ND	ND	ND	ND
Actin, muscle OS = *Manduca sexta* PE = 2 SV = 1	ACT_MANSE	42,149	ND	ND	213/15	559/15	ND	ND	ND	ND	ND
Selenium-binding protein 1 OS=*Homo sapiens* GN=SELENBP1 PE = 1	SBP1_HUMAN	52,928	ND	ND	160/4	ND	113/3	108/3	ND	232/10	142/4
Retinal dehydrogenase 1 OS=*Homo sapiens* GN = ALDH1A1 PE = 1 SV = 2	AL1A1_HUMAN	55,454	ND	ND	254/6	ND	ND	ND	ND	121/3	ND
Lactotransferrin OS=*Sus scrofa* GN = LTF PE = 1 SV = 3	TRFL_PIG	79,514	ND	ND	126/7	ND	ND	ND	111/7	103/5	ND
Keratin, type II cytoskeletal 1 OS=*Homo sapiens* GN=KRT1 PE = 1	K2C1_HUMAN	66,170	ND	ND	ND	296/12	298/9	ND	ND	ND	ND
Phosphoglycerate mutase 1 OS=*Bos taurus* GN=PGAM1 PE = 2	PGAM1_BOVIN	28,948	ND	ND	ND	113/4	ND	ND	ND	ND	ND
Actin, cytoplasmic 1 OS=*Bos mutus* grunniens GN = ACTB PE = 2	ACTB_BOSMU	42,064	ND	ND	ND	ND	248/18	ND	ND	ND	ND
Elongation factor 1-alpha, somatic form OS = *Xenopus laevis* GN = eef1as PE = 2	EF1A0_XENLA	50,524	ND	ND	ND	ND	142/6	ND	ND	ND	ND
Retinal dehydrogenase 1 OS=*Bos taurus* GN = ALDH1A1 PE = 1 SV = 3	AL1A1_BOVIN	55,398	ND	ND	ND	ND	128/2	ND	ND	ND	ND
Lactoperoxidase OS=*Homo sapiens* GN = LPO PE = 1 SV = 2	PERL_HUMAN	81,149	ND	ND	ND	ND	341/11	424/13	ND	ND	ND
Actin-17 OS=Dictyostelium discoideum GN = act17 PE = 3	ACT17_DICDI	41,773	ND	ND	ND	ND	ND	177/10	ND	ND	ND
Elongation factor 1-alpha OS=Blastobotrys adeninivorans GN = TEF PE = 3	EF1A_BLAAD	50,426	ND	ND	ND	ND	ND	115/4	ND	ND	ND
Polymeric immunoglobulin receptor OS=*Homo sapiens* GN=PIGR PE = 1	PIGR_HUMAN	84,429	ND	ND	ND	ND	ND	105/3	109/6	ND	ND
Actin-1 OS = *Aedes aegypti* GN = ACT-1 PE = 2 SV = 2	ACT1_AEDAE	42,045	ND	ND	ND	ND	ND	ND	490/17	361/20	335/17
Keratin, type II cytoskeletal 6A OS = *Rattus norvegicus* GN=Krt6a PE = 1 SV = 1	K2C6A_RAT	59,555	ND	ND	ND	ND	ND	ND	331/7	123/3	ND
Ceruloplasmin OS=*Homo sapiens* GN=CP PE = 1	CERU_HUMAN	122,983	ND	ND	ND	ND	ND	ND	196/6	188/8	124/3
Alpha-enolase OS=*Bos taurus* GN = ENO1 PE = 1 SV = 4	ENOA_BOVIN	47,639	ND	ND	ND	ND	ND	ND	163/7	171/6	ND
Gelsolin OS=*Homo sapiens* GN = GSN PE = 1 SV = 1	GELS_HUMAN	86,043	ND	ND	ND	ND	ND	ND	148/6	186/8	122/5
Elongation factor 1-alpha OS=Onchocerca volvulus PE = 2	EF1A_ONCVO	51,090	ND	ND	ND	ND	ND	ND	106/3	ND	ND
Alpha-crystallin B chain OS=*Bos taurus* GN=CRYAB PE = 1	CRYAB_BOVIN	20,024	ND	ND	ND	ND	ND	ND	ND	140/3	ND
Annexin A1 (Fragment) OS = *Gallus gallus* GN = ANXA1 PE = 2 SV = 1	ANXA1_CHICK	14,446	ND	ND	ND	ND	ND	ND	ND	121/2	ND

*ND* not detected; or score less than 100

These proteins were identified in the tear of healthy cats, before infection with *T. gondii* cysts, 5 days and 21 days later, analyzed in triplicate (a, b and c)

**Fig. 3:**
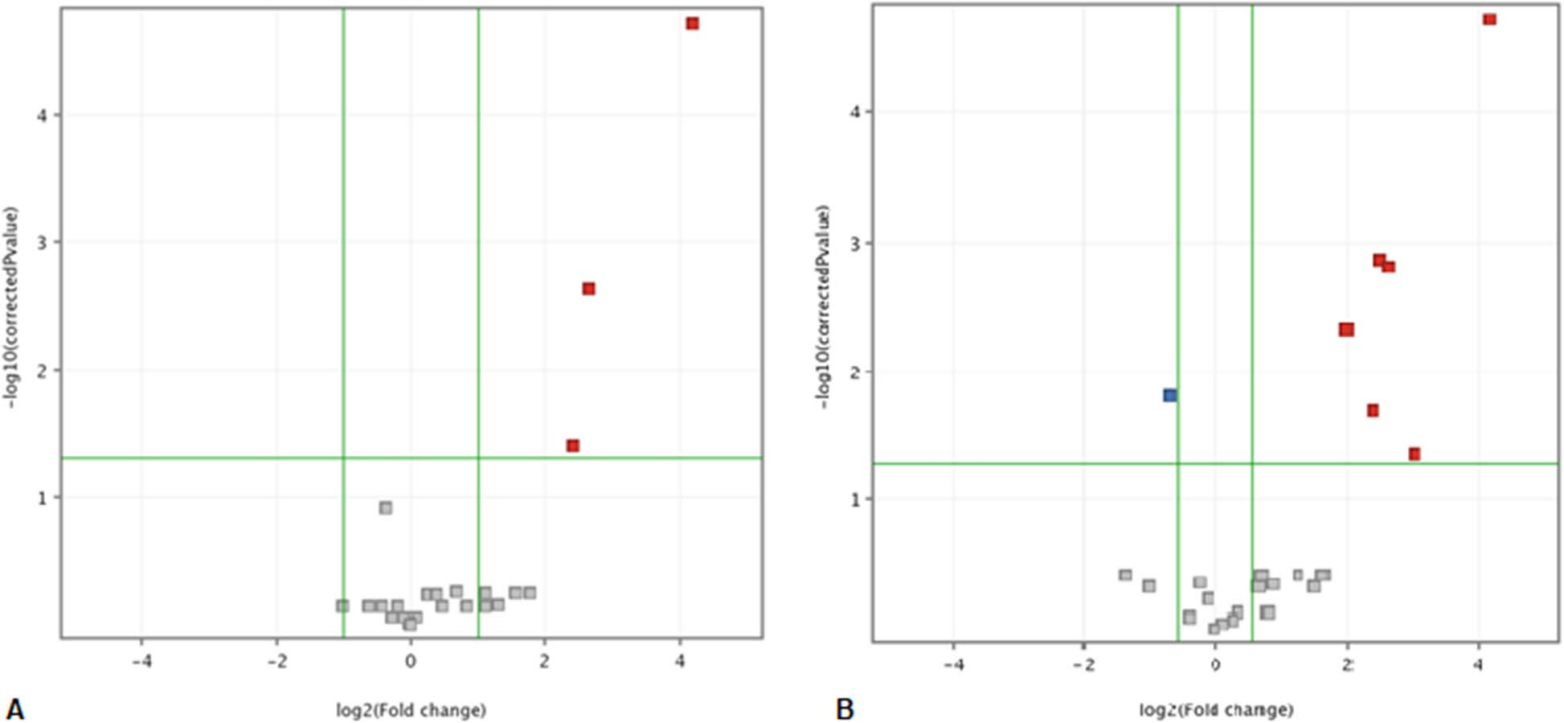
Abundance of proteins. Points above the horizontal line had significant abundance (t-test, *p* < 0.05). **A** Comparison between days D0 and D21. Proteins that have positive accumulation are represented by red dots (*fold-change* = 1.5). **B** Comparison between days D5 and D21. Proteins that had negative and positive accumulation are represented by blue and red dots, respectively (*fold-change* = 1.5)

Table 1 shows the abundance of each protein in each group. There was no significant difference (Student t-test, *p* ≤ 0.05) between the proteins found when comparing their abundance between D0 and D5. There was a statistical difference between D0 and D21 for the following proteins: ACT1_AEDAE (actin), CERU_HUMAN (ceruloplasmin) and GELS_HUMAN (gelsolin), all more abundant on D21; these proteins were above the fold change (Fig. 3A). Regarding D5 and D21, there was a significant difference (Fig. 3B) for: KV1_CANLF (kappa immunoglobulin), LAC_PIG (lambda immunoglobulin), TRFL_PIG (lactotransferrin), ACT1_AEDAE (actin), CERU_HUMAN (ceruloplasmin) and GELS_HUMAN (gelsolin), all more abundant on D21, in addition to OVOS2_HUMAN (ovostatin), more abundant on D5.

**Fig. 4:**
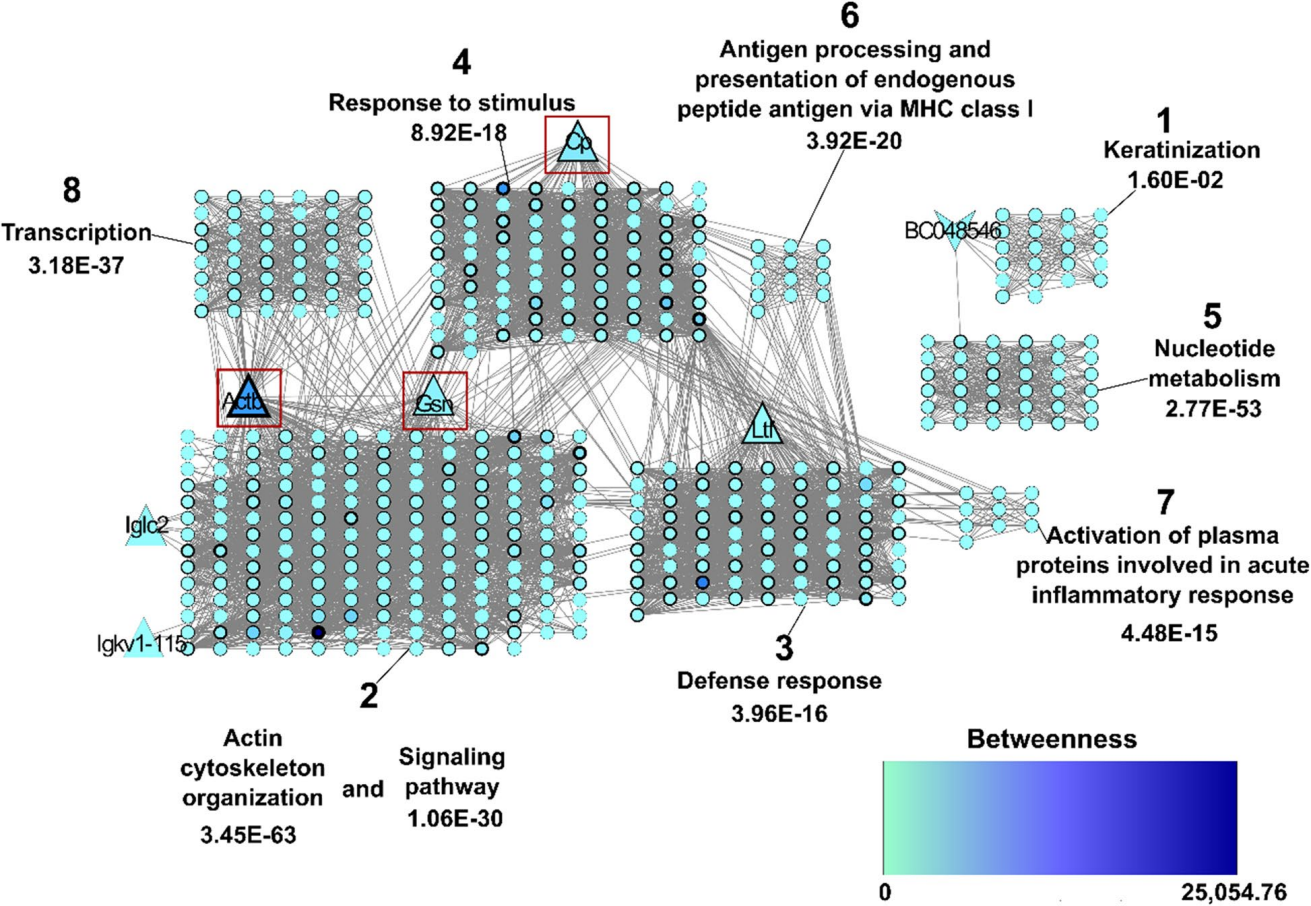
Interaction network. Proteins from *M. musculus* homologous to those identified in *F. catus* as differentially abundant on day 21 after inoculation with *T. gondii*. Each node represents a protein. The larger triangle-shaped nodes denote the proteins identified as having increased abundance, and the inverted triangle-shaped proteins represent those that were identified as having reduced abundance in comparison with the proteins identified on day 5. The proteins within the red square also had a significant difference of expression on day 21 after inoculation compared to proteins identified on day 0. The node degree is represented by the edge of the nodes, where wider edges correspond to greater node degrees. The betweenness value is represented on the blue tone scale. Clusters are numbered and separated into grids. One or two biological processes were assigned to each cluster. The *p*-value of the hypergeometric test with Benjamini & Hochberg false discovery rate (FDR) correction is shown for each process

The protein interaction network was constructed from *Mus musculus* proteins homologous [17] to those identified as differentially abundant in the *F. catus* TF (Table 2) on day 21 after inoculation with *T. gondii*. The network had a total of 490 nodes (proteins), 5782 connectors and eight clusters (Fig. 4).

**Table 2:** *Mus musculus* proteins homologous to the proteins identified in the cats’ TF

*Mus musculus*		*Felis catus*					
Protein name in network^**a**^	N° Acession^**b**^	Protein Name^**c**^	Expression^**d**^	% Identity^**e**^	BN^**f**^	H^**g**^	CL^**h**^
Iglc2	P01844	Ig lambda chain C region	Up	61.5	Y	N	2
BC048546	Q3UU35	Ovostatin homolog 2	Down	60.1	Y	N	1
Actb^i^	P60710	Actin-1	Up	96.0	Y	Y	2
Cp^i^	Q61147	Ceruloplasmin	Up	83.2	Y	Y	4
Gsn^i^	P13020	Gelsolin	Up	91.9	Y	Y	2
Igkv1–115	ENSMUSP00000132003	Ig kappa chain V region GOM	Up	61.0	Y	Y	2
Ltf	P08071	Lactotransferrin	Up	64.0	N	Y	3

^a^*Names of mus musculus* proteins found to be homologous to those identified in *F. catus* tears and shown in the network (Fig. 4)

^b^Accession number of *M. musculus* proteins homologous to those identified in *F. catus* tears and shown in the network (Fig. 4)

^c^Proteins identified as differentially expressed in *F. catus* tears 21 days after infection compared to those on D0 and D5

^d^Indicates whether the protein was up- or down-expressed in *F. catus* tears 21 days after infection compared to those on D0 and D5

^e^Percentage of identity between homologs of *M. musculus* and *F. catus*

^f^Indicates whether the protein is considered a bottleneck (Y) or not (N), depending on whether its betweenness value is equal to or greater than the average

^g^Indicates whether the protein is considered a Hub (Y) or not (N), taking into account whether its node degree value is equal to or greater than the average

^h^Indicates the cluster to which the protein belongs, as shown in Fig. 4

^i^Proteins differentially expressed in *F. catus* tears 21 days after infection compared to those identified at time 0

For each cluster, a genetic ontology enrichment analysis was performed, of which nine biological processes were assigned to the clusters, including: signaling pathway; defense and stimulus response; antigen processing; antigen presentation of endogenous peptide via class I MHC; and activation of plasma proteins involved in the acute inflammatory response. The expression level of each process can be related to the expression level of the protein identified in *F. catus* present in the respective cluster (Table 2).

Among the differentially identified proteins, actin-1 had the highest betweenness and node degree values (Additional file 1).

## Discussion

The most abundant proteins in human TF are lipocalin, lysozyme, lactoferrin and serum albumin [18, 19]. Studies of animal TF have also been carried out [2, 11–15]. However, although there are studies of the tear proteome of healthy cats [17], none has been published so far on the protein expression of the TF of cats infected with *T. gondii*.

The results of this study demonstrate similarity between the tear components of domestic cats, humans [20] and other animal species, such as rabbits [11], which presented lactoferrin, albumin and lipocalin among the major proteins. Additionally, the results obtained by Shamsi et al. (2011) [2], who evaluated and compared the TF of humans, cows, sheep and camels, indicated similarity between the most predominant proteins in the species they studied (lactoferrin, serum albumin, lipocalin, lysozyme), some of which were also abundant in the samples evaluated by us. In the present study, proteins never described in the tear film of any species were also identified, such as allergen Fel d 4; major allergen 1 and elongation factor 1-alpha.

Regarding the most abundant proteins identified in the TF of the cats evaluated in this study, a brief discussion of the main functions of each in the ocular system is presented in Table 3. Those differently expressed (*p* ≤ 0.05) in animals after infection with *T. gondii* will be discussed in more detail.

**Table 3:** Most abundant proteins identified in the TF of the evaluated cats and their respective functions.

Protein	Function
Allergen Fel d 4	Cat-specific allergen lipocalin, which composes two of the ten feline allergens found in their body fluids and secretions, although not yet described in tears. Responsible for allergic reactions of other mammals (especially humans) to felines. Homologous to the main equine allergen, Equ c1. Its biological function in felines remains unknown [48–50].
Major allergen 1	Major feline allergen, responsible for up to 90% of the IgE-mediated response in allergic reactions to cats. Its biological function in felines, like lipocalin Feld 4, remains unknown. Since it has not yet been described in the tears of domestic cats, it is another possible source triggering allergic processes to felines, as well as Fel d 4 [48–51].
Serum albumin	One of the 1500 proteins already reported in tear film of several species. It acts in local antimicrobial defense, as a marker of the integrity of the blood-ocular barrier and maintenance of tear osmolarity [2, 11, 15, 18, 33, 52, 53].
Aldehyde dehydrogenase	It had been reported in human tear samples. Some subfamilies of this enzyme are present in the cornea and lens and are part of the defense mechanism against injuries caused by ultraviolet rays, maintaining the integrity of these structures. In addition, they help maintain cellular homeostasis, as they protect cells against reactive oxygen species, and consequently from oxidative stress, as well as protect the lens from cataract formation [20, 54, 55].
Lactoperoxidase	An enzyme present in several mammalian bodily fluids, such as tears, with main antimicrobial activity. Furthermore, it protects cells against peroxidative effects and carcinogens [56].
Inhibitor of carbonic anhydrase	It has already been reported in several bodily fluids, such as tears, including those already described in the eye system of dogs. It binds to and inhibits some isoforms of carbonic anhydrase. Anhydrase, in turn, helps maintain the pH of the TF, in addition to protecting the corneal epithelium against aggression [57, 58].
Deleted in malignant brain tumors 1 protein	A glycoprotein that can bind to mucosal defense proteins, such as IgA, lactoferrin and albumin, participating in the innate immune response. Since it also binds to the C1q fraction of complement, it indirectly participates in the response to pathogens, which is important for the inflammatory response against invading microorganisms. It has already been described in the TF of humans [20, 59].
Haptoglobin	Its main function in the body is to bind to hemoglobin dimers that are released into the circulation after hemolysis. Furthermore, it is part of the acute phase proteins, thus participating in immunomodulation. In humans there is an increase in infectious diseases such as conjunctivitis [60].
Glutathione S transferase	Acts as an antioxidant in the eye system [61, 62].
Polymeric immunoglobulin receptor	This glycoprotein has already been described in tear samples of humans. It is crucial for the effectuation of humoral and cellular immune responses and for the elimination of antigens, since it is through its connection that several biological responses are performed, such as phagocytosis, capture of immune complexes and release of inflammatory mediators [20, 63].
Retinal dehydrogenase 1	It helps to preserve the integrity of vision, as well as minimizing the effects of oxidative stress, acting as an antioxidant for the cornea [55, 64].
Immunoglobulin heavy variable	The most abundant heavy-chain immunoglobulin in the TF is IgA, along with lysozyme, lactoferrin, and lipocalin. Its main function is to defend the ocular mucosa against pathogens [32, 65].
Selenium-binding protein	It acts to regulate oxidative stress and is secreted by the lacrimal gland. Studies in humans who have keratoconjunctivitis sicca indicate that the reduction of selenium expression by the lacrimal glands leaves the cornea of these patients exposed to oxidative damage [3, 66].
Alpha-enolase	Glycolytic enzyme expressed in most cells, important in autoimmune diseases, cancer and fungal diseases. Studies analyzing the tear film in humans with dry eye syndrome revealed an increase in this protein in affected patients [67].
14–3-3 protein sigma	A family of different isoforms of proteins secreted by the cornea and conjunctiva, important for the regulation of metabolism and cell cycle, as well as for apoptosis, protein transport, and transcription [4].
Malate dehydrogenase	Although not secreted by the lacrimal gland, this enzyme can be released into the TF after damage to the corneal and conjunctival epithelium, even by collection using the Schimmer test. It is important for cell transduction and metabolism [68–70].
Serotransferrin	An important glycoprotein for the structural integrity of the epithelial cells that make up the ocular system, as well as protection against pathogens [71].
Keratin	It is present in the epithelial and myoepithelial cells that make up the lacrimal and meiosis glands, interacting with the lipid layer of the tear film [72].
Phosphoglycerate mutase	In mammalian tissue, type B can be found in the retina. In a proteomic study of retinal samples of myopia in guinea pigs, it was observed that of the retinal proteins expressed in myopic eyes, the phosphoglycerate mutase was one of those that presented a reduction in its expression when compared to the control group [73]. According to the authors, it is important for cell metabolism and regulation of biological processes [73, 74].
Elongation factor 1-alpha	A binding protein, essential for protein synthesis, also acting in the regulation of actin cytoskeleton, protein degradation and apoptosis, in addition to being reported as a promoter of viral replication [75, 76].
Alpha cristallin A, B	Most abundant protein components in mammalian eye lenses, helping to maintain lens transparency; present in the cells of the lacrimal gland ducts. Mutations in these can cause cataracts. Furthermore, they also protect against oxidative stress and high temperatures. Alpha B present in the TF may originate from corneal degeneration or from the epithelial cells of the conjunctiva [77, 78].
Annexin A1	Regulating/mediating glucocorticoid protein with anti-inflammatory action. In research carried out with rats, it was concluded that annexin also has anti-inflammatory action on ocular inflammation, especially in cases of uveitis. Studies carried out with humans concluded that it is physiologically present in tears [79–81].
Actin	Cell cytoskeleton component [24].
Gelsolin	Important for cell locomotion and phagocytosis. Responsible for cell differentiation, epithelial cell regeneration and apoptosis. Expressed in all tissues of the ocular system, secreted by TF [26, 27].
Ceruloplasmin	Studies indicate there is high affinity interaction with lactoferrin [29].
Ig Kappa; Ig Lambda	Immunoglobulin components of the immune response, present in the cornea, probably derived from the serum [30].
Lactotranferrin	Anti-inflammatory and antimicrobial activities and an important role in innate immunity, helping to fight pathogens and maintain the health of the eye system; also acts to protect eye tract cells against oxidative damage [3, 33, 34].
Ovostatin	Important metalloproteinase for the degradation and reabsorption of extracellular matrix components [36].

Although the strain of *T. gondii* used to infect the animals in this study was ME49, classified as moderately virulent in mice [21, 22], the results suggest that it was able to modify the expression of some proteins. In this sense, studies have shown that a high parasite load of a type II strain, such as ME49, is able to stimulate high levels of cytokines (in response to infection) similar to strain I, with greater virulence [22]. Additionally, according to Angeloni (2013) [23], this strain was able to stimulate the immune response through the secretion of pro-inflammatory cytokines, although at lower intensity than the strain with greater virulence (RH), in infected trophoblast cells. These findings indicate that even being a less virulent strain, not associated with clinical ophthalmic signs [22], ME49 is able to stimulate the immune response, in line with the results of the present study, which revealed changes in the tear proteome after infection of animals, suggesting its ability to penetrate ocular cells. It should also be noted that the susceptibility of the affected species also influences the appearance of signs associated with infection. In general, regardless of the strain, immunocompetent cats tend to have asymptomatic evolution after infection with *T. gondii* [6], which may also explain why the animals in this study did not show clinical signs.

Regarding the proteins that showed a significant difference when their expressions on D21 were compared to D0, we suggest that the increase in actin, a component of the cytoskeleton of cells, is associated with the entry and persistence of *T. gondii* in cells of the ocular system of infected cats. This hypothesis is based on the results of a study that demonstrated that not only factors inherent to the parasite, but also components of the host, can facilitate the entry of *T. gondii* into cells of its tissues. Among these, the microtubules and microfilaments present in the host tissue stand out [24]. The authors concluded that 24 host proteins are involved in the entry of *T. gondii* into the host cell, of which six act by modifying the dynamics of the actin cytoskeleton, leading to its increase in the host cell’s periphery and thus facilitating the entry of the parasite, which in the present case possibly occurred in the cells of the ocular system. This reorganization of the cytoskeleton is represented in cluster 2 of the network, where actin is found. These findings help explain why actin was more strongly expressed in infected animals in the present study, since *T. gondii* can parasitize cells of the ocular system [6, 7]. Furthermore, in the network, actin is a hub and bottleneck protein due to the large number of connections that cross it and because it is a meeting point of two clusters, one of them represented by the transcription regulation process. It has been shown that an actin-based myosin engine is associated with transcription of ribosomal genes in the cell nucleus [25], suggesting that actin may also participate in regulating the expression of genes involved in *T. gondii* infection.

Gelsolin, in turn, is a modulating protein present in the actin filament, acting in its remodeling, which is important for cell locomotion and phagocytosis. It is responsible for cell differentiation, epithelial cell regeneration and apoptosis, and is expressed in all tissues of the ocular system and is secreted in the TF. It is more abundant in tissues belonging to the ocular surface compared to parenchymal organs [26, 27]. As mentioned above, since actin can be modified to facilitate the entry of *T. gondii* into host cells, possibly the increase in gelsolin was associated with this. The relationship between actin and gelsolin was represented in the network, since both proteins were within the same cluster (cluster 2).

Regarding ceruloplasmin, it is a ferroxidase found in plasma [28], about which studies indicate a high-affinity interaction with lactoferrin [29], which (as previously described) is one of the most abundant tear film proteins [2, 18]. We suggest that its high affinity with lactoferrin was a secondary cause of its increase in the samples evaluated, since lactoferrin showed increased expression on D21 compared to D5, as discussed below. In the interaction network, ceruloplasmin belonged to cluster 4, associated with the response to stimuli, with a close relationship with the cluster in which lactoferrin is located, of the defense immune response. Thus, the results of the network corroborate the analysis and interpretation of the data presented here.

Also with regard to D21, there was a significant difference in expressions, in addition to actin, ceruloplasmin and gelsolin, of lactotransferrin and kappa and lambda immunoglobulins, when compared to D5 samples. Ig kappa and Ig lambda are light chain immunoglobulins, components of the immune response, which also make up the cornea, where their concentration is related to the serum concentration of IgG. These immunoglobulins are probably derived from serum, since their levels in tear and aqueous humor are not high [30]. These immunoglobulins belong to cluster 2 of the network, related to the signaling mechanism. We suggest that the increased expression of these proteins comes from the immune response of infected cats, since there is an IgG response in animals infected with *T. gondii*, indicative of recent seroconversion or chronicity [31].

Lactotransferrin is produced by acinar cells of the main and accessory lacrimal glands. It is one of the proteins with the strongest antimicrobial action in the tear film, together with lipocalins, lysozyme and IgA [32]. Within the network, this protein belongs to cluster 3, represented by the defense response process. The increase in its expression on D21 can be explained by its anti-inflammatory and antimicrobial activities and important role in innate immunity, helping to fight pathogens and maintain the health of the ocular system [33, 34]. Furthermore, lactotransferrin protects eye tract cells against oxidative damage [3], and cells producing reactive oxygen species have anti-toxoplasma activity [35]. Thus, it is possible that these cells acted to combat *T. gondii* and generated reactive species, responsible for oxidative damage, which triggered the increase in lactotransferrin.

Still regarding the comparison between the proteins observed on D21 in relation to D5, ovostatin was more abundant on D5. This protein is a metalloproteinase (MMP1, 3, 8), of the zinc-dependent endopeptidase family, important for the degradation and reabsorption of extracellular matrix components. Like other metalloproteinases, ovostatin is important for biological processes such as angiogenesis, morphogenesis and tissue repair [36]. In the interaction network, the homolog of this protein is related to clusters 1 and 5, represented by the processes of keratinization and nucleotide metabolism. Niehus et al., (2012) [37] reported that *T. gondii* components stimulate human macrophages to synthesize metalloproteinase, which would act in the degradation of the cell matrix collagen, facilitating parasite migration through tissues. Our research results corroborate the study cited above, since ovostatin is a metalloproteinase and its level was high in the acute phase of infection by *T. gondii* in the samples evaluated.

There was no significant difference in the expression of proteins from D5 samples compared to D0 due to the time required for the parasite, after infection of intestinal cells, to spread through the bloodstream and reach the cells of the ocular system, generating a response on the proteomic expression. A study with a murine model found that ocular alterations occurred in mice 15 days after infection with *T. gondii*, strain ME49 [38]. Thus, further elucidative studies are needed in this regard.

Additionally, although trypsin was identified in the samples evaluated, we suggest its presence wass due to the fact it was used during the preparation of tears for proteomic analysis.

## Conclusions

From the results obtained, it was possible to identify 54 proteins present in the tear film of healthy domestic cats infected with *T. gondii* with a score equal to or greater than 100. Of these, 37 were components expressed in healthy animals. Most of the identified proteins are part of the ocular surface’s defense system against injuries. The most strongly expressed proteins in animals in the chronic phase of *T. gondii* infection are associated with the immune response to the parasite. Furthermore, an increase in actin was also observed, a protein that was possibly modified by the agent to facilitate its entry into the cells of the ocular system of infected cats.

It is important to know the proteins present in the TF of healthy domestic cats, as well as those affected by some pathology, since they can be used both as early biomarkers of diseases as well as for monitoring disease progress, as is already the case in humans and other animal species. Although other body fluids are already used for this purpose, TF is a good option, especially because its monitoring is noninvasive and it is easier to collect in felines compared to blood samples. Since this study was a pioneer in the proteomic evaluation of the tear film of cats infected with *T. gondii*, future studies should be carried out to better elucidate the use of the protein profile not only in cats affected by toxoplasmosis, but also by other diseases of clinical importance, a such as glaucoma, keratoconjunctivitis sicca, diabetes and numerous neoplasms. New studies can reveal more comprehensive results and enable the identification of effective biomarkers for diseases in felines, allowing better treatments.

## Methods

### Screening of animals

Twelve mixed breed domestic cats (*Felis catus*), of both sexes, were included in the study at 30 days of age and were monitored in catteries by the research team during the subsequent months. The animals were donated by the owners for research after formal consent. The selected animals were dewormed upon admission, immunized against the main infectious diseases of felines at 3 months of age, and castrated between 7 and 8 months of age. At approximately 15 months of age, the cats were infected with tissue cysts of *T. gondii*. After the end of the study, all animals were sent for adoption.

Before being infected, these animals were screened, which included physical examination, blood count, serum biochemistry, serology and nested polymerase chain reaction (PCR) for FIV/FeLV; serology for *T. gondii*; and coproparasitological examination. The last two exams were performed every 15 days, from the admission of the cats until the infection with *T. gondii*, with all results being negative in both cases. Thus all the animals included in the study were negative for *T. gondii* and FIV/FeLV, and were healthy at the end of the study.

The project was approved by the Ethics Committee on the Use of Animals (CEUA), under protocols 003/17 and 024/15. All procedures were conducted in accordance with the Association for Research in Vision and Ophthalmology’s (ARVO) Statement for the Use of Animals in Ophthalmic and Vision Research and the NIH Statement and also followed the Cat-Friendly Practice guidelines.

Blood counts were performed using an automated hematology counter (ABC Vet, automated blood cell counter, Horiba, Kyoto, Japan). Commercial kits and a biochemical analyzer (BIO-2000 IL, Bioplus Produtos para Laboratórios Ltda, Barueri, SP) were used to measure levels of alanine aminotransferase, aspartate aminotransferase, gamma-glutamyltransferase, bilirubin, urea and serum creatinine.

For the diagnosis of FIV/FeLV, 5 ml of venous blood was obtained from puncture of the jugular vein, of which 2.5 ml was stored in tubes without ethylenediamine tetraacetic acid (EDTA) and allocated for performance of the commercial enzyme-linked immunosorbent assay (ELISA) serological test (FIV Ac FeLV Ag Test Kit), according to the manufacturer’s recommendations. The remaining 2.5 ml was stored in tubes with EDTA and submitted to genomic DNA extraction, using the Easy-DNA kit (Invitrogen®), according to the manufacturer’s recommendations. To perform nested PCR, primers and the methodology previously described were used [39–41].

For the investigation of antibodies against *T. gondii* IgM and IgG, indirect immunofluorescence reaction was used, developed according to the description by Pinto et al. (2009) [42].

### *Toxoplasma gondii* infection

A total of 26 female Swiss mice were used, weighing between 20 and 25 g, which received 40 oocysts of *T. gondii* strain ME49 (provided by professor João Luís Garcia, from State University of Londrina). This strain is considered to have moderate virulence and is not associated with clinical ophthalmic signs [21, 22]. The animals were maintained with commercial feed and water ad libitum and observed daily for 6 weeks. After these 6 weeks of infection, the animals were euthanized in a CO_2_ chamber. Then, to confirm the infection, their brains were removed and a fragment of each was evaluated (squashed) for the presence of *T. gondii* cysts, based on their morphology. These cysts were observed in all samples evaluated, and were quantified in a Neubauer chamber. Subsequently, the brains were homogenized and 800 *T. gondii* cysts were offered to each cat, orally, after 24-h fasting.

### Coproparasitological analysis to confirm infection

After ingestion of the infected mice brains, for the coproparasitological evaluation of the infection phase, feces of the cats were collected daily, from the 3rd day until 30 days after infection. The samples were processed and examined by the modified centrifuge-flotation technique with sucrose solution, described by Sheather (1923) [43] and modified by Duszynsk and Wilber (1997) [44]. To identify the eliminated oocysts, stool samples were processed according to Dubey (2001) [45] and Gondim et al. (2002) [46].

### Identification of phases after infection and tear collection

Tear samples were collected on days 0 (before infection), 3, 5, 7, 9, 11, 13, 15, 17, 19 and 21 (after infection). However, for proteomic analysis, in addition to the samples from day 0, those corresponding to days 5 and 21 after infection were selected for later comparison of results. The choice was based on the analysis of coproparasitological results, from which it was observed that peak of oocyst release in the feces occurred on day 5, characterizing the acute phase of the infection, as indicated by Galvão et al., (2014) [47]. After day 14, there was no further release of oocysts in the feces. Thus, we selected the sample on day 21, 7 days after the total absence of oocyst release in the feces, characterizing the beginning of the chronic phase.

Feline tear collection was performed with Schirmer’s Tear Test 1 kit (TLS-1). For this purpose, the standardized paper strip (5 mm notch) was inserted into the ventral conjunctival sac of both eyes and the length of the moistened portion was measured with a millimeter scale immediately after the protocol time (60 s). Subsequently, the wet strips obtained from the 12 cats were transferred to 1.5 mL Eppendorf tubes and kept under refrigeration for 2 h. Then, the samples were centrifuged in a refrigerated centrifuge at 4 °C, to avoid evaporation, for 10 min, at 15000 xg, and were stored in three pools of supernatants: one pool of 12 samples from day 0 (D0), one from day 5 (D5) and one from day 21 (D21), which were frozen in triplicate, at a temperature of − 20 °C, until the moment of proteomic analysis. The samples were quantified by the method of Bradford (1976), using BSA as a standard.

### Proteomic analysis

#### Enzymatic digestion with trypsin

The frozen tear samples were processed at the National Biosciences Laboratory (LNBio), belonging to the National Center for Research in Energy and Materials (CNPEM), located in the city of Campinas, São Paulo.

Approximately 2 μL were pipetted from each sample pool (D0, D5 and D21). To start the digestion process, 22 μL of H_2_O was added to each sample pool to obtain a final volume of approximately 25 μL. The reactions were made in triplicate. Subsequently, 25 μL of urea and 0.5 μL of dithiothreitol (DDT) 0.5 mol L^− 1^ were added to each sample, which was incubated for 25 min at 56 °C. Then, 1.4 μL of 0.5 mol L^− 1^ iodoacetamide was added, and again incubated for 30 min at room temperature in a place protected from light. A volume of 0.5 μL of 0.5 mol L^− 1^ DDT was added to the samples, which were incubated for 15 min at room temperature in a place protected from light. The samples were diluted by adding 131 μL of 50 mmol L^− 1^ ammonium bicarbonate, 1.83 μL of CaCl2 and 3 μL of 20 ng μL^− 1^ trypsin. Between each addition and incubation, the tubes were shaken for 5 s and centrifuged for 10 s. The samples were incubated at 37 °C for 16 h (overnight) and later acidified (pH below 2) to stop the action of trypsin, by adding 4 μL of 100% formic acid.

#### Sample desalination

Once the acidification of the sample with a reagent tape was proven, the desalination process was carried out. For this process, the samples were added in stage-tips that received the addition of 100 μL of 100% methanol and 100 μL of 0.01% formic acid.

After several centrifugation steps, the stage-tips were inserted into new tubes (1.5 mL), with the addition of 100 μL of a solution of 20% ultrapure water + 80% acetonilate + 0.1% formic acid.

After centrifugation, the stage-tips were removed from inside the tubes and subjected to drying in a speed vac. Afterwards, the samples were analyzed with a Q-Tof-Premier mass spectrometer.

#### Mass spectrometry

For protein analysis, a 2.0 μL aliquot of peptides resulting from digestion with the protein was separated in RP-nanoUPLC (nanoAcquity, Waters) C18 column (100 × 100 mm) coupled to a Q-Tof Premier mass spectrometer (Waters) with nanoelectrospray source at a flow rate of 0.600 ul min^− 1^. The gradient was 2–90% acetonitrile in 0.1% formic acid over 60 min. Voltage was set at 3.5 kV, cone voltage at 30 V and source temperature at 100 °C. The instrument was operated in “top three” mode, in which an MS spectrum was acquired followed by MS/MS of the three most intense peaks detected. After MS/MS fragmentation, the ion was placed on the exclusion list for 60 s, and for the analysis of endogenous cleavage peptides, real-time exclusion was used. Samples were injected in triplicate.

#### Data analysis

For data analysis, spectra were acquired with the help of MassLynx v.4.1 software and raw data files (RAW files) were converted into a peak list format (mgf) by the software Mascot Distiller v.2.6.2.0, 2009 (Matrix Science Ltd). Subsequently, the data were compared against the general UniProt database, using the Mascot Daemon engine v.2.3.2 (Matrix Science Ltd), with carbamidomethylation as fixed modification and methionine oxidation as variable modification, loss of a trypsin cleavage, and 0.1 Da tolerance for ion precursors and fragments. The file merging was performed in triplicate. Proteins with a score equal to or greater than 100 were considered for analysis. The justification for the choice was based on the fact that protein scores are significant values pre-established by the bioinformatics tool that considers the most likely protein. Thus, the higher the score obtained, the lower the probability that the analysis is a random result. The results were analyzed using the Student t-test, with the Agilent Mass Profiler Professional 15.1 software, considering a significance of *p* ≤ 0.05 of the protein abundance between days D0 and D5, D0 and 21, and D5 and D21. In addition, the days of protein accumulation were plotted and separated by a principal component analysis (PCA) graph.

### Systems biology

A network was built from *Mus musculus* proteins homologous to those identified in *Felis catus* as differentially abundant. The proteins were subjected individually to interactome analysis using the STRING version 11.0 database (http://string-db.org) with the following parameters: meaning of network edges: confidence; active interaction sources: text mining, experiments, databases, co expression, neighborhood, gene fusion and co-occurrence; minimum required interaction score: high confidence (0.700); max number of interactors to show: 1st and 2nd shell: no more than 50 interactions. The file for each network was downloaded in TSV format and later the files were merged and analyzed using the Cytoscape software version 3.8.2. The modularity and centrality properties (betweenness and node degree) of the network were calculated using the igraph package of the statistical tool R. For each cluster, an enrichment analysis of gene ontology was performed using the BiNGO version 3.0.4 plugin.

In the network, proteins are represented by nodes. The node degree (number of connections that cross the node) and betweenness (a node’s ability to join two or more clusters) properties were calculated. Proteins with an above-average degree of knotting are called hubs and proteins with an above-average betweenness are called bottlenecks. Both hub proteins and bottleneck proteins play an important regulatory role within the network.

## Supplementary Information

**Additional file 1.**

## Data Availability

Most data generated or analyzed during this study are included in this article. The other data are available from the corresponding author on request.
